# Characterization of forebrain neurons derived from late-onset Huntington's disease human embryonic stem cell lines

**DOI:** 10.3389/fncel.2013.00037

**Published:** 2013-04-05

**Authors:** Jonathan C. Niclis, Anita Pinar, John M. Haynes, Walaa Alsanie, Robert Jenny, Mirella Dottori, David S. Cram

**Affiliations:** ^1^Monash Immunology and Stem Cell Laboratories, Monash UniversityClayton, VIC, Australia; ^2^The Florey Institute of Neuroscience and Mental Health, University of MelbourneParkville, VIC, Australia; ^3^Monash Institute of Pharmaceutical Sciences, Monash UniversityParkville, VIC, Australia; ^4^Department of Anatomy and Neuroscience, Centre of Neuroscience Research, University of MelbourneParkville, VIC, Australia

**Keywords:** Huntington's disease, human embryonic stem cells, neuronal differentiation, GABAergic neurons

## Abstract

Huntington's disease (HD) is an incurable neurodegenerative disorder caused by a CAG repeat expansion in exon 1 of the Huntingtin (HTT) gene. Recently, induced pluripotent stem cell (iPSC) lines carrying atypical and aggressive (CAG60+) HD variants have been generated and exhibit disparate molecular pathologies. Here we investigate two human embryonic stem cell (hESC) lines carrying CAG_37_ and CAG_51_ typical late-onset repeat expansions in comparison to wildtype control lines during undifferentiated states and throughout forebrain neuronal differentiation. Pluripotent HD lines demonstrate growth, viability, pluripotent gene expression, mitochondrial activity and forebrain specification that is indistinguishable from control lines. Expression profiles of crucial genes known to be dysregulated in HD remain unperturbed in the presence of mutant protein and throughout differentiation; however, elevated glutamate-evoked responses were observed in HD CAG_51_ neurons. These findings suggest typical late-onset HD mutations do not alter pluripotent parameters or the capacity to generate forebrain neurons, but that such progeny may recapitulate hallmarks observed in established HD model systems. Such HD models will help further our understanding of the cascade of pathological events leading to disease onset and progression, while simultaneously facilitating the identification of candidate HD therapeutics.

## Introduction

Huntington's disease (HD) is an autosomal dominant neurodegenerative disorder caused by an expanded CAG repeat tract in exon 1 of the *HTT* gene (Group, 1993). Disease alleles (CAG_>35_) exhibit an age-dependent penetrance, with the lowest disease range (CAG_36−39_) associated with a later onset (McNeil et al., 1997). CAG_40+_ alleles are associated with full penetrance, while larger expansions (CAG_60+_) result in juvenile or infantile onset (Andrew et al., 1993; Squitieri et al., 2006). Substantial correlation of ~50% exists between CAG repeat length and age of onset, with secondary contributions from known and unknown genetic and environmental factors (Andrew et al., 1993; Wexler et al., 2004). HD affects approximately 1 in 10,000 individuals worldwide, with onset of disease usually in the fourth or fifth decade of life. HD culminates in death 15–20 years after persistent, irreversible and debilitating clinical symptoms (Naarding et al., 2001). To date, there is no cure for HD and consequently, there is an unmet clinical need for more effective therapeutics.

The *HTT* gene comprises 67 exons and encodes a ubiquitously expressed protein of approximately 350 kDa called Huntingtin (HTT). HTT plays a critical role in early embryogenesis with homozygous *HTT* null mouse embryos exhibiting incomplete neural development and lethality at embryonic day 8.5 and 10.5 of gestation (Zeitlin et al., 1995). HTT has been extensively studied in relation to its interacting partners, sub-cellular localization and effects on gene expression, and acts in a variety of cellular systems (Zuccato et al., 2010). The clinical symptoms of HD seen in patients can largely be classified as either neural, such as behavior and cognitive alterations, or motor, such as involuntary movements and abnormalities of voluntary movements. Underlying neurodegeneration is most prominent within the GABAergic medium spiny neurons of the striatum although widespread neuronal loss occurs with disease progression (Graveland et al., 1985; Vonsattel et al., 1985).

Understanding the molecular nature of HD is critical to the development of novel and efficacious therapies. Numerous animal models such as the R6/2 transgenic mouse (Mangiarini et al., 1996) together with post mortem patient tissues have proven valuable resources for identifying and elucidating major CNS cellular mechanisms and hallmarks that contribute to HD pathology; these are comprehensively reviewed in Zuccato et al. (2010) and include transcriptional dysregulation, CAG repeat expansion, excitotoxic stress, autophagy-lysosomal, and proteasome-ubiquitin system perturbation, anterograde and retrograde transport interference, mitochondrial dysfunction and cholesterol biosynthesis alteration.

The hierarchical relationship between the disparate mechanisms/hallmarks and their degree of individual contribution to the overall HD phenotype still remains uncertain. New human HD models such as pluripotent stem cells (PSCs) may provide an alternative system to shed light on this etiological question. Studies investigating hPSC lines, specifically human embryonic stem cell (hESC) lines, carrying pathogenic CAG repeat mutations have provided limited data in relation to the consequences of disease allele expression (Niclis et al., 2009; Bradley et al., 2011; Seriola et al., 2011). More recently, mouse and human induced pluripotent stem cell (iPSC) lines have been analysed for the appearance of HD hallmarks (Camnasio et al., 2012; Castiglioni et al., 2012; HDIPSCC, 2012; Jeon et al., 2012) and several phenotypes have been observed, including transcriptional dysregulation, CAG repeat instability, mutant HTT aggregates, cholesterol biosynthesis perturbation, lysosomal dysfunction and neuronal vulnerability. However, phenotypes reported from the various HD iPSC studies differ significantly and rarely correlate, for example CAG repeat instability is seen in human but not R6/2 iPSCs. A further caveat is that the HD iPSC lines studied to date either carry rare homozygous HD mutations or alleles with egregious repeat tracts (CAG_60−180_) indicative of juvenile and infantile onset and may represent atypical disease scenarios.

Limited studies assess the appearance of HD phenotypes in hPSC lines with clinically relevant trinucleotide expansions (CAG_35−60_) that correlate with late ages of onset. To this end, we have extended an earlier study by Niclis et al. (2009) using two HD hESC lines SI-187 (CAG_51/19_) and SI-186 (CAG_37/15_) that were generated from embryos identified with pre-implantation genetic detection (PGD). Here we show that the HD hESC lines are indistinguishable from control hESCs with respect to pluripotent characteristics, mitochondrial function, forebrain neural differentiation capabilities, relevant cellular phenotypes and with no evidence of a dysregulated transcriptome in key HTT associated genes. However, the larger of the two CAG repeat cell lines showed an altered responsiveness to the neurotransmitter glutamate. Overall, these results are informative for establishing a human neuronal cellular model of HD.

## Materials and methods

### Culture and neural differentiation of hESC lines

Two HD hESC lines SI-186 (CAG_37/15_) and SI-187 (CAG_51/19_) (Niclis et al., 2009) as well as two wildtype control hESC lines, HES3 and H9 (Thomson et al., 1998; Reubinoff et al., 2000) were used. All hESC lines were screened for karyotypic abnormalities using Geisma staining and found to maintain euploid 46 XX karyotypes (data not shown). All four hESC lines were grown as bulk cultures on γ-irradiated mouse embryonic fibroblasts (MEFs) according to previously described conditions (Costa et al., 2008); briefly, cells were grown in DMEM/ F-12, supplemented with 0.1 mM β-mercaptoethanol, 1% non-essential amino acids, 1% Glutamax, 25 U/ml penicillin, 25 U/ml streptomycin, and 20% knockout serum replacement (all Invitrogen).

hESC neural differentiation was performed as previously described (Song et al., 2011a). Briefly, ~3000 hESCs were distributed to each well of a round-bottom ultra-low attachment 96-well plate (Corning) containing 100 μl of Differentiation Medium (Neurobasal A, 5% ITS-X, 2.5% Penicillin/Streptomycin, 5% Glutamax, 5% B27 and 5% N2; Invitrogen). From d0–d21 cells were grown as neurospheres at 37°C in 5% CO_2_ in air, with 0.125% polyvinyl alcohol and 10 μM Roh-Associated Coil Kinase (ROCK) inhibitor Y-27632 at d0–4. From d0–d21 media was supplemented with 20 ng/ml epidermal growth factor (EGF) and 20 ng/ml fibroblast growth factor (FGFb) (R&D Systems), and 100 ng/ml of Noggin (R&D Systems) from d0–8. Neurospheres were passaged at d14 by manual sectioning in half. To promote neuronal differentiation neurospheres were plated at d21 in Differentiation Medium onto 24-well plates (BD Biosciences) coated with 20 μg/ml poly-D-lysine and 20 μg/ml laminin (Invitrogen) in the absence of growth factors and media changed every 4 days for up to 24 days.

### Flow cytometry

Cells were harvested using TrypLE Select (Invitrogen), passed through a 70 μm filter and 1 × 10^5^ cells stained with various antibodies listed in Table 1. Samples were analysed using a BD Canto II flow cytometer with data analyses performed using GateLogic. All samples were gated to assess only single cells as determined by forward scatter area vs. height channels and live cells as determined by negative DAPI selection. Background fluorescence was subtracted using unlabeled cells. A total of 50,000 events (~40,000 live cells) were recorded, compensations were performed using single label antibody controls; positive gates were designated on concentration-matched isotype control antibodies.

**Table 1:** **Antibodies and dilutions utilized for flow cytometry and immunocytochemistry**.

**Antibody target**	**Species**	**Conjugate**	**Dilution**	**Company**
CD9	Mouse	FITC	1:30	BD
TRA-1-60	Mouse	APC	1:25	BD
FORSE-1	Mouse	N/A	1:1000	DSHB
CD56	Mouse	PerCPcy5.5	1:50	BD
β-III-TUBULIN	Mouse	N/A	1:500	Millipore
Huntingtin	Rabbit	N/A	1:100	Millipore
SOX2	Goat	N/A	1:200	ABcam
OTX2	Rabbit	N/A	1:1000	Millipore
GABA	Rabbit	N/A	1:500	Millipore
FOXG1	Rabbit	N/A	1:50	ABCam

### Genotyping

Genotyping was performed as previously described (Niclis et al., 2009). Briefly, HU3 and HU4 primers (Sermon et al., 1998) were used to amplify the CAG repeat expansion region in exon 1 of *HTT*. The HU4 sense primer was labeled with 6-carboxyfluorescein (6-FAM) and sizing of amplicons performed with an ABI Prism 3100 DNA sequencer coupled with GeneScan Software according to previously described methods (Cram et al., 2000). The number of CAG repeats was accurately calculated to ±1 bp from GeneScan sized F-PCR products, using the formula: CAG repeat number = (size of PCR product – size of non-CAG repeat region)/3.

### Immunocytochemistry

Neurospheres were collected at d10 or 21 and fixed for 15 min at room temperature with 4% paraformaldehyde, cryoprotected in sucrose (20% w/v in 0.1 M PBS) and embedded in OCT compound (Tissue–Tek) before serial sectioning (6 × 10 μm thick sections) on a cryostat (Leica). Neuronal cultures were fixed at d35 or 45 for 15 min at room temperature with 4% paraformaldehyde, permeabilized with 1% Triton X-100 (Ameresco), blocked using 10% normal donkey serum (Invitrogen) and incubated with primary antibodies described in Table 1 at 4°C overnight. Species-specific AlexaFluor (Invitrogen) or Dyelight (Jackson Laboratories) 488 and 555 secondary antibodies (all 1:200) were added for 2 h at room temperature and then counterstained with 0.1 μg/ml DAPI (SIGMA). Coverslips were mounted onto glass slides using Fluorescent Mounting Media (DAKO). Bound fluorescence was detected using an Olympus BX51 microscope coupled to the ULH100HG fluorescence system. Images were captured using a DP70 camera.

### Cell growth and viability assays

Duplicate MEF coated T25 culture flasks were seeded with 1 × 10^6^ hESCs for each line. Cells were harvested at 24 and 72 h. FACS analysis of DAPI, CD9, and TRA-1-60 staining was used to distinguish live hESCs (DAPI–/CD9+/TRA1–60+) and MEFs (DAPI–/CD9–/TRA1–60–). Doubling rates were calculated based on hESC and live cells only, according to the following formulae: [Log_2_(β/α)]/2; α = 24 h count and β = 72 h count. Viability assays were performed similarly on hESCs 24 h after passage but with DAPI live and dead cell gating performed by FACS after CD9 and TRA-1–60 gating. Neurosphere growth rates were determined by measuring area sizes using Adobe Photoshop CS5, the average value for 12 neurospheres equated a single replicate, with three averaged values analysed at each time point for each line. Data values at d21 were doubled to account for passaging in half of spheres at d14. Data was statistically analysed with PRISM, using a One-Way ANOVA with a Kruskal–Wallis Test and Dunn's post test. Means were graphed with the SEM and a *p*-value (^*^) of < 0.05 considered significantly different.

### Neuronal functional analysis

Neurons were cultured as described above. For intracellular Ca^2+^ [(Ca^2+^)_i_] imaging, neurons were loaded with Fluo4-AM (2 μM, for 45 min at 37°C, as previously described (Watmuff et al., 2012). Media was then replaced with HEPES buffer (of composition, mM, NaCl 145; MgSO_4_ 1; KCl 5; glucose 10; CaCl_2_ 2.5; HEPES 10; pH 7.4) containing bovine serum albumin (0.3% w:v) and placed on a heated (37°C) stage. Neurons were viewed using a Nikon A1R confocal microscope and excited with a 488 nm laser, emission was recorded at 525/50 nm at two image frames per second. After 2 min of baseline recording glutamate (30 μM) or vehicle was added to each well and imaging continued for 90 s as previously described (Raye et al., 2007; Khaira et al., 2011; Watmuff et al., 2012). After this time KCl (30 mM) was added to each well and imaging continued for a further 60 s. For analysis, background emission light was subtracted from analysis regions defined within each cell body and emission intensity within each region calculated using Nikon Elements software. The maximal elevation of intracellular [Ca^2+^]_i_ in response to vehicle or glutamate is expressed as the net fraction of the KCl-induced maximal increase in [Ca^2+^]_i_.

### Quantitative real-time PCR

Total RNA was extracted from ~ 1×10^6^ cells using the commercially available RNeasy Mini Kit (Qiagen) as per the manufacturer's instructions and treated with RNase-Free DNase (Qiagen). RNA was quantitated using a ND-1000 Spectrophotometer (NanoDrop Technologies). Purified RNA (0.4–2 μg) was reverse transcribed to single stranded cDNA with SuperScript III reverse transcriptase using the First Strand Synthesis System Kit (Invitrogen) and treated with RNase H to remove contaminating RNA.

cDNA products were subjected to qRT-PCR of selected genes (Table 2) using TaqMan® Universal PCR Master Mix and the inventoried TaqMan® Gene Expression Assay Kit (Applied Biosystems) according to manufacturer's recommendations. Glyceraldehyde 3-phosphate dehydrogenase (*GAPDH*) was used for normalization of RNA input, two independent reactions were prepared for each sample. Reaction plates were run on the 7500 Real Time PCR System (Applied Biosystems) at the following thermocycler conditions: stage 1, 50°C for 2 min; stage 2, 95°C for 10 min and stage 3, 40 cycles of 95°C for 15 s and 60°C for 1 min. The relative quantification (RQ) of gene expression in each sample was analysed using SDS Software version 1.3 (Applied Biosystems). The Ct value for each sample was measured in duplicate and the average normalized against the endogenous control *GAPDH* to determine the ΔCt value. The ΔCt values were then standardized against the calibrator's ΔCt (wildtype HES3 line) to yield the ΔΔCt. The RQ was then calculated as 2^−ΔΔCt^. Statistical analysis for qRT-PCR data was performed using GraphPad Prism with a one-way ANOVA analysis of each gene at each specific time point. A Kruskal–Wallis test was performed on the non-matched, non-parametric data. The *p*-value was set to <0.05 and a Dunn's post-test performed with RQ data from three culture replicates plotted as a mean and scale bars denoting the mean ± SEM.

**Table 2:** **TaqMan gene expression assay details used for qRT-PCR**.

**Gene symbol**	**Amplicon length (bp)**	**Gene bank number**	**Assay ID**
GAPDH	93	NM_002046.3	Hs99999905_m1
HTT	66	NM_002111.6	Hs00918134_m1
PGC1a	74	NM_013261.3	Hs01016719_m1
DRP1	88	NM_005690.3	Hs00247147_m1
BDNF	116	NM_170733.3	Hs00380947_m1
DRD2	64	NM_000795.3	Hs00241436_m1
PENK	56	NM_001135690.1	Hs00175049_m1
SREBP1	90	NM_001005291.2	Hs01088691_m1
β-III-TUBULIN	82	N/A	Hs00964967_g1

*All primers were purchased from Applied Biosystems*.

### Western blotting

1 × 10^6^ cells were lysed in 500 μl of Cell Lysis Buffer (Cell Signaling) and protein concentrations determined using a BCA assay (Pierce, Thermo Scientific). Protein samples (10 μg) in 1× SDS sample buffer containing 10% β-mercaptoethanol were electrophoresed on 4–12% NuPAGE gels (Invitrogen). Proteins were transferred to immobilon PVDF transfer membranes (Millipore), blocked in TBS-T containing 5% non-fat dry milk powder and then immunoblotted with either monoclonal antibody 1HU-4C8 (Millipore, 1:2000) specific for epitopes within amino acids 181–810 on wildtype and mutant HTT or monoclonal antibody 5TF1-1C2 (Millipore, 1:5000) which has a high avidity for long polyglutamine epitopes (>15 polyglutamine residues). A secondary anti-mouse HRP antibody (Calbiochem) in combination with an ECL kit was finally used to illuminate HTT protein.

### JC-1 mitochondrial staining

JC-1 a cationic dye that undergoes a fluorescence emission shift from green (529 nm) to red (590 nm) upon accumulation in the mitochondrial matrix (Simeonova et al., 2004), was employed to measure changes in the mitochondrial membrane potential (MMP). Undifferentiated hESCs (1 × 10^6^) prepared from bulk cultures were incubated in media containing 2 μM of JC-1 for 30 min, washed twice in PBS and then analysed using a BD FACS Canto II flow cytometer 488 nm excitation and emission detection dually from both FITC and PE channels. Compensations were performed with hESC specific CD9 and EpCAM antibodies, conjugated to FITC and PE respectively. Data analysis was conducted using FACS Diva software (BD).

## Results

### Characterization of pluripotent HD hESC lines

**Figure 1:**
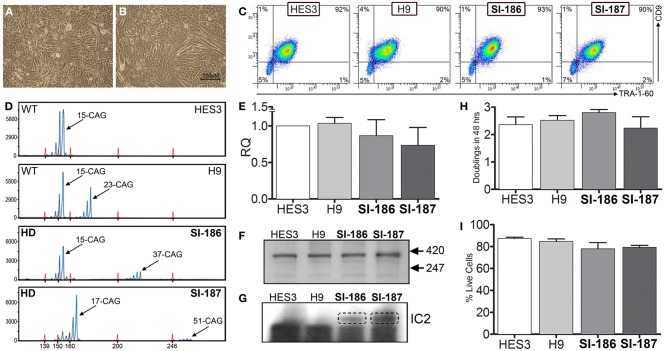
**Characterization of pluripotent HD hESC lines demonstrates identical characteristics to control lines. (A,B)** Brightfield morphology of hESC bulk cultures. **(C)** Flow cytometry analysis of hESCs seen as a TRA-1-60+/CD9+ population; feeder MEFs are a distinct CD9–/TRA-1-60-sub-population. **(D)** FL-PCR representation of HD genotypes. **(E)** qRT-PCR of HTT mRNA expression levels. **(F)** Western blot of an N-terminal epitope antibody on the 350 kDa HTT protein. **(G)** Western blot of hESC lines immunoprobed with the IC2 monoclonal antibody. **(H)** Growth rates of hESC lines (One-Way ANOVA, *n* = 3, *p* ≥ 0.05) and **(I)** cell viability comparisons (One-Way ANOVA, *n* = 3, *p* ≥ 0.05).

HD and control hESC lines were originally derived as colony cultures that intrinsically comprise significant heterogeneity with differential degrees of pluripotency and differentiation capacities across a gradient of isolatable sub-fractions (Laslett et al., 2007; Hough et al., 2009; Kolle et al., 2009). To eliminate this stochasticity, HD hESC lines were adapted to an enzymatically passaged bulk culture system to provide homogenous cultures amenable to stringent molecular and functional comparative evaluations. Of note, adaptation rates of HD lines were commensurate with controls, requiring ~3–5 passages, and produced morphologically identical bulk culture monolayers of hESCs with low cytoplasmic-to-nuclear ratios (Figures 1A,B). Robust homogeneity was achieved with hESC bulk culture conditions, evidenced by a tightly clustered population of TRA-1-60+/CD9+ pluripotent cells and an absence of single positive cells indicating that the hESCs were maintained in a pluripotent state (Figure 1C). Both HD hESC lines exhibited FACS profiles with pluripotent markers similar to control lines verifying stable undifferentiated states (Figure 1C). Further, no significant differences in cell growth rates (doublings/48 h) were observed between HD and control hESC lines (SI-187 2.24, SI-186 2.8, HES3 2.36, and H9 2.52, *p* > 0.05; Figure 1H) or cell viability during enzymatic passaging (SI-187 79.3%, SI-186 78.0%, HES3 84.7%, and H9 87.3%, *p* > 0.05; Figure 1I).

Analysis of FL-PCR products confirmed correct CAG repeat genotypes for HD lines SI-187 (CAG_51/19_) and SI-186 (CAG_37/15_) and control lines HES3 (CAG_15/15_) and H9 (CAG_23/15_) (Figure 1D). qRT-PCR using primers specific for conserved sequences on both wildtype and mutant alleles confirmed *HTT* mRNA was expressed at similar levels in HD and control hESC lines (Figure 1E). Western blot analysis using a polyclonal antibody reactive to an N-terminal epitope on the 350 kDa HTT protein (Figure 1F) confirmed HTT protein expression in all four hESC lines. Further immunoprobing using the IC2 monoclonal antibody that specifically reacts with long polyglutamine epitopes >15–20 residues (Trottier et al., 1995) detected a ~350 kDa band exclusively within SI-187 and SI-186 cells confirming mutant HTT expression in both HD lines (Figure 1G).

### Neural differentiation is unperturbed by an expanded CAG tract

**Figure 2:**
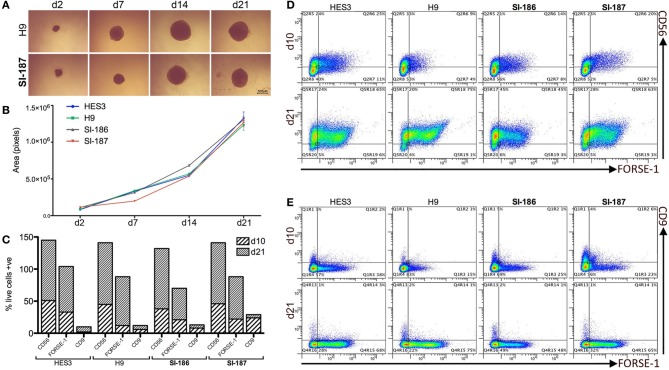
**Neural progenitor lineage specification and expansion is consistent between HD and control neurospheres. (A)** Brightfield time course representations throughout neurosphere differentiation stages **(B)**. Neurosphere growth rate analysis across all HD and control lines (One-Way ANOVA, *n* = 3, *p* ≥ 0.05) from an initial 3000 cell seeding density. Flow cytometry quantification of antigens CD9, CD56, and FORSE-1 at differentiation days, d10 and d21 showing **(C)** absolute single antibody expression levels and **(D)** co-expression scatter plots for CD56 and FORSE-1 and **(E)** CD9 and FORSE-1.

HD neurodegeneration emerges within the forebrain and progresses to whole-brain atrophy. Differentiation of HD hESC lines to a regionally relevant forebrain phenotype was achieved using the spin aggregation neurosphere system we have previously reported (Song et al., 2011a). Seeding 3000 hESCs per well resulted in morphologically similar neurospheres within 24 h and throughout the neurosphere differentiation stage between HD and control lines (Figure 2A). Neural growth rates were equivalent between HD and control lines indicating the presence of mHTT does not interfere with neural precursor division and differentiation (*p* > 0.05; Figure 2B).

Quantitative flow cytometric assessment throughout neurosphere stages demonstrated highly analogous expression profiles across HD and control lines. All lines progress early to a neural fate and near total expression (>90%) of the pan-neural marker CD56 is observed by d21 (Figure 2C). Neural differentiation exhibited an anterior identity with >50% of cells expressing the forebrain surface antigen FORSE-1 (Figure 2C). The forebrain population was confirmed as neural in character with >90% of the FORSE-1+ population co-staining with CD56 by d21 (Figure 2D), and all FORSE-1+ cells separating from minor CD9+ fractions (Figure 2E). All hESCs rapidly exit a pluripotent state with low levels of the hESC marker CD9 d10 which falls to ~2% by d21 (Figure 2E). Intriguingly, a delay in CD9 downregulation is observed in the fully penetrant SI-187 line at d10, although by d21 CD9 expression is consistent across all lines (Figure 2E). Overall, these findings suggest that pathogenic HD mutations do not perturb neural progenitor generation, expansion or specification to an anterior identity.

**Figure 3:**
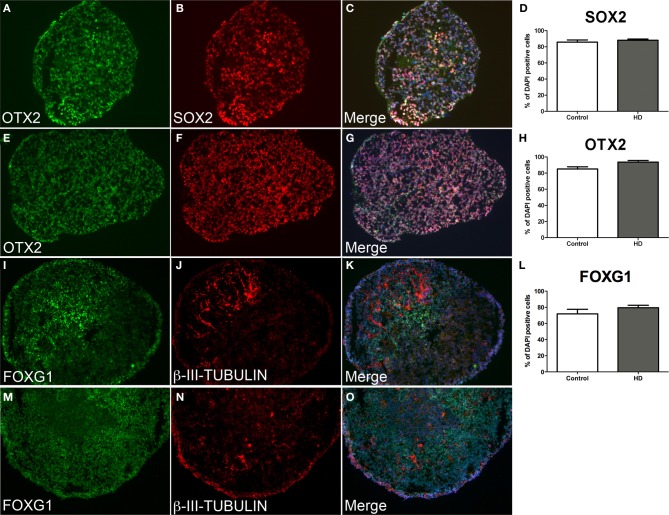
**Equivalent neurosphere neural progenitor gene expression and forebrain specification from HD and control cryosectioned immunostained neurospheres.** Neurospheres at d10 of differentiation from HD line SI-187 **(A–C)** and control line H9 **(E–G)**, demonstrate robust expression of the telencephalic marker OTX2 (green) and similarly the neural progenitor marker SOX2 (red). Merged images demonstrate co-expression of OTX2 and SOX2 and quantitative evaluation shows no significant difference between control and HD neurospheres (**D, H**, paired *t*-test, *n* = 4 *p* ≥ 0.05). Neurospheres at d21 of differentiation from HD line SI-187 **(I–K)** and control line H9 **(M–O)** show extensive expression of the forebrain transcription factor FOXG1 (green) and nascent expression of the cytoskeletal marker β-III-TUBULIN (red) which predominates within FOXG1 expressing regions, with no quantitative differences between control and HD neurospheres (**L**, paired *t*-test, *n* = 8 *p* ≥ 0.05).

Control and HD neurospheres exhibited equivalent progenitor phenotypes at d10 (Figures 3A–H). Strong expression of the multipotent neural stem cell transcription factor SOX2 was observed in control (85.8%) and HD (88.2%) cells equally (Figure 3D, data not significant *p* = 0.05). This was concomitant with a rostral fate seen by the robust expression of the telencephalic marker OTX2 in comparable proportions between control (88.0%) and HD (92.4%) cells (Figure 3H, data not significant *p* = 0.05), and by co-localization of both proteins (Figures 3C,G). Importantly, continued differentiation to d21 facilitated robust expression of the forebrain specific transcription factor FOXG1 within the majority of neurosphere cells in both control and HD sections (Figures 3I–O). Expression levels of FOXG1 were not significantly different between control (71.9%) and HD (79.4%) neurospheres (Figure 3L, *p* = 0.05). Limited numbers of neuronal cells were seen at the end of neurosphere culture, detected by β-III-TUBULIN expression (Figures 3J,N). FOXG1 expressing regions preferentially co-localize with β-III-TUBULIN expression indicating maturation of anterior cells to a post-mitotic neuronal fate (Figures 3K,O).

**Figure 4:**
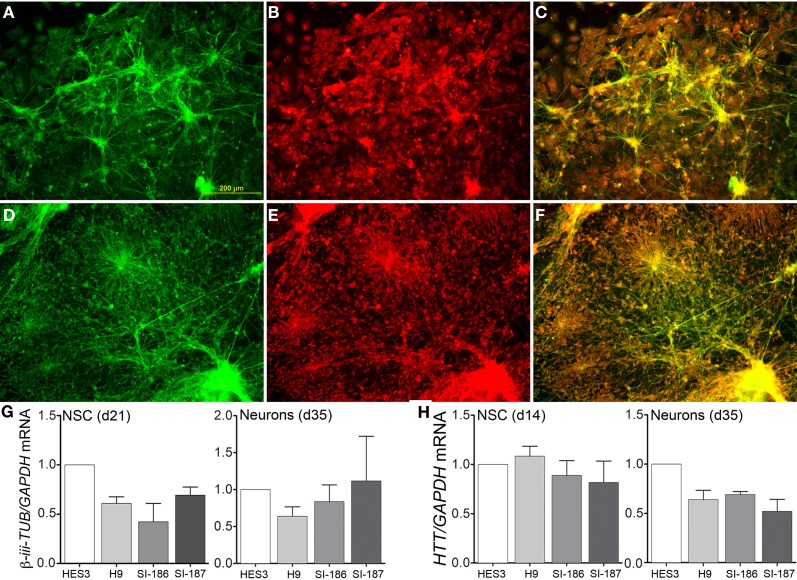
**Unperturbed neuronal differentiation in HD lines.** Neuronal differentiation of **(A–C)** control HES3 and **(D–F)** HD SI-187 cells at d35, with immunocytochemical staining demonstrating **(A,D)** β-III-TUBULIN expression (green) and **(B,E)** HTT expression (red). **(C,F)** Merged images reveal HTT localization within neuronal cells. **(G)** Neuronal differentiation qRT-PCR comparisons of β-iii-TUBULIN expression during late neurosphere (d21) and neuronal (d35) stages. **(H)** HTT transcript expression across HD and control lines during mid-neurosphere (d14) and neuronal (d35) stages.

**Figure 5:**
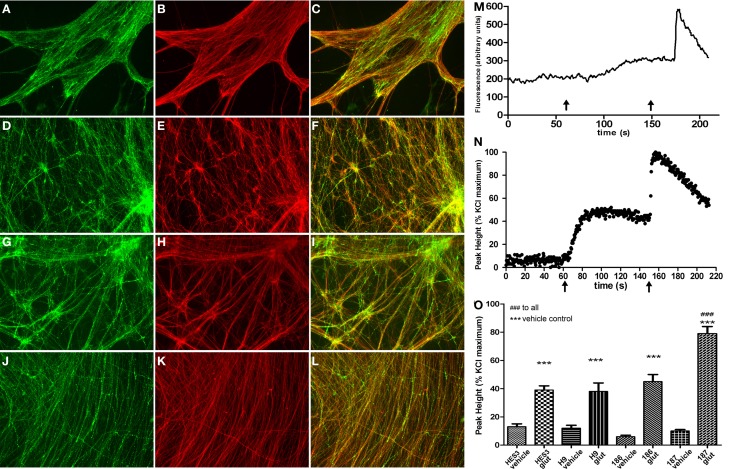
**Efficient differentiation to GABAergic neurotransmitter subtypes and glutamate response perturbations in HD neurons. (B,E,H,K)** Neuronal differentiation extended to d45 generated elaborate networks shown by β-III-TUBULIN (red) immunostaining. GABAergic neurons detected by GABA immunostaining (green) were equivalently detected throughout neuronal cultures from control lines **(A)** HES3 and **(D)** H9, as well as HD lines **(G)** SI-186 and **(J)** SI-187. **(C,F,I,L)** Merged images illustrating co-localization between GABA and β-III-TUBULIN. **(M)** Example of a typical raw trace after background subtraction measured in arbitrary units of fluorescence intensity and **(N)** a typical neuronal Ca^2+^ response profile, left *x*-axis arrows indicate glutamate (30 μM) addition and right *x*-axis arrows KCl (30 mM) addition. **(O)** Intracellular [Ca^2+^]_i_ levels in response to vehicle and glutamate (30 μM) addition for all control and HD neuronal cultures.

Subsequent to neurosphere plating on extracellular matrices, neuronal differentiation disseminated throughout cultures in control (Figure 4A) and HD neurons (Figure 4D) as shown by cytoskeletal β-III-TUBULIN immunostaining. Quantitative validation was made of these observations during late neurospheres (d21) and neuronal (d35) differentiation stages by β-*III-TUBULIN* transcript expression comparisons (Figure 4G). Antibody staining against an N-terminal HTT epitope revealed cytoplasmic and nuclear protein localization (Figures 4B,E) and specifically preferential nuclear reactivity within the nuclei of neurons with no discernable differences between control (Figure 4C) and HD neurons (Figure 4F). Further, quantitative *HTT* transcript expression levels were equal across all HD and control lines during neural precursor and neuronal differentiation stages (Figure 4H). Crucially, neuronal differentiation of anterior neurosphere cells culminated in robust detection of the neurotransmitter GABA, which was observed in equal proportions throughout the elaborate neuronal networks produced from control HES3 (Figures 5A–C) and H9 (Figures 5D–F) as well as HD SI-186 (Figures 5G–I) and SI-187 (Figures 5J–L) cell lines. Co-staining for β-III-TUBULIN and GABA demonstrate the majority of neuronal cells possess a GABAergic identity (Figures 5C,F,I,L).

### Functional responses of neuronal cultures

Neurons derived from this culture protocol were compared to their respective vehicle control wells with all four hESC derived neuronal cultures responding to glutamate (30 μM) with a significant elevations of [Ca^2+^]_i_, Figure 5M for a typical raw trace after background subtraction and Figure 5N for a typical response profile (One-Way ANOVA with *post-hoc* Tukey test). Interestingly, neurons derived from the fully penetrant SI-187 cell line responded to glutamate with a significantly greater elevation of [Ca^2+^]_i_ than neurons from the wildtype HES3 and H9 lines, or the partially penetrant SI-186 line (*p* < 0.05, One-Way ANOVA with *post-hoc* Tukey test, Figure 5O).

### Transcriptional analysis of mutant HTT modulated genes

**Figure 6:**
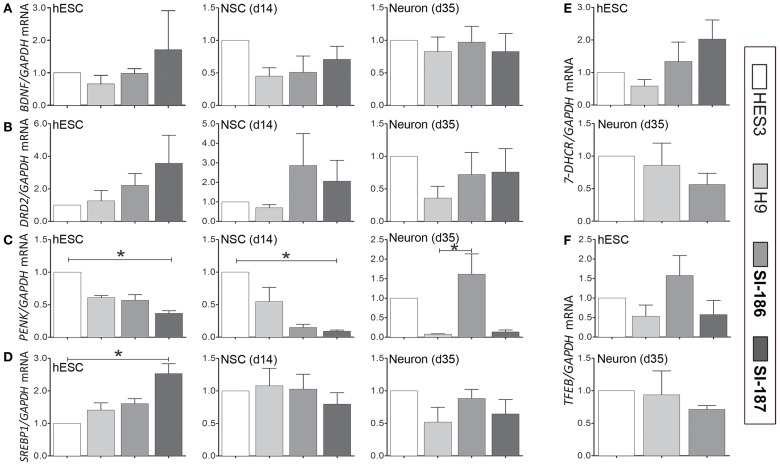
**qRT-PCR transcriptional analysis of relevant HD related transcripts modulated by mutant HTT.** Relative transcript quantification for all four lines are normalized to the control HES3 line and were evaluated across a differentiation time course at the undifferentiated stage (d0), mid-neurosphere stage (d14) and neuronal differentiation stage (d35). Transcripts are assessed for **(A)** *BDNF*, **(B)** *DRD2*, **(C)** *PENK*, **(D)** *SREPB1*, **(E)** *7-DHCR*, and **(F)** *TFEB*. One-Way ANOVA, ^*^p < 0.05.

Recent studies of HD human embryonic carcinoma cells (hECCs) and iPSC lines demonstrate *in vitro* gene expression alterations (Gaughwin et al., 2011; Castiglioni et al., 2012). Whether similar transcriptome disruptions are evident in late-onset CAG length hESC lines remains uncertain. Expression levels of critical transcripts identified in previous studies and those with direct paths from mHTT expression to gene disruption were assessed by qRT-PCR in undifferentiated HD and control lines (day 0), within neurospheres derivatives at day 14 and at neuronal promoting stages (day 35) (Figure 6). Selected for assessment were neural-specific genes Brain-Derived Neurotrophic Factor (*BDNF*; Zuccato and Cattaneo, 2007, 2009), dopamine receptor D2 (*DRD2*) and proenkephalin (*PENK*) (Luthi-Carter et al., 2000; Dunah et al., 2002; Hodges et al., 2006). Also assessed were the cholesterol biosynthesis transcripts 7-Dehydrocholesterol reductase (*7-DHCR*) and sterol regulatory element-binding protein 1 (*SREBP1*) (Valenza et al., 2005; Valenza and Cattaneo, 2011), as well as transcription factor EB (*TFEB*) a master regulator of lysosomal biogenesis (Sardiello et al., 2009; Settembre et al., 2011; Camnasio et al., 2012). At day 0 and 14 significant downregulation of *PENK* mRNA expression was observed in SI-187 cells compared to HES3 cells (*p* < 0.05; Figure 6C) and *SREBP1* was significantly up-regulated in SI-187 compared to HES3 at day 0 (*p* < 0.05; Figure 6D). Nevertheless, significant gene expression changes in *PENK* and *SREBP1* mRNA of HD SI-187 cells were not corroborated when compared to the second control line H9 (*p* > 0.05), suggesting these discrepancies are a consequence of interline variation as opposed to CAG repeat length. mRNA expression levels of the remaining genes *BDNF, DRD2, 7-DHCR7*, and *TFEB* were similar across HD and control lines at days 0, 14, and 35 (*p* > 0.05) (Figures 6A,B,E,F).

### Assessment of mitochondrial gene expression and function

**Figure 7:**
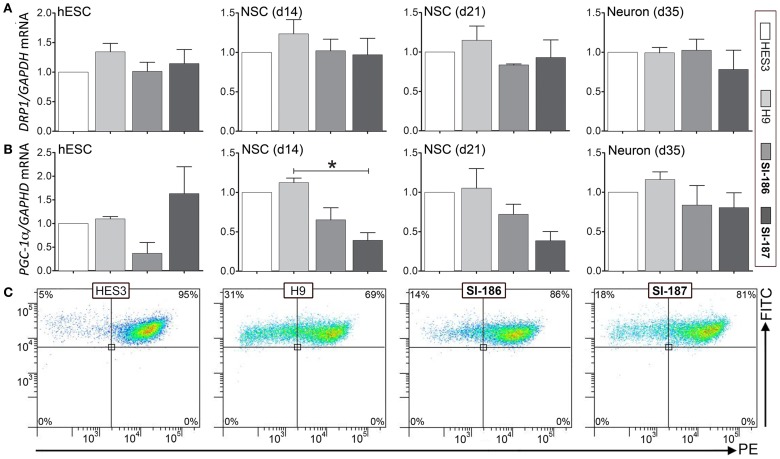
**Assessment of mitochondrial gene expression and function.** Relative transcript quantification for all four lines are normalized to the control HES3 line for mitochondrial genes **(A)** *DRP1* and **(B)** *PGC-1*α at the undifferentiated (d0), neurosphere (d14) and neuronal (d35) stages of differentiation. **(C)** Mitochondrial functional JC-1 assay quantifying activity of the mitochondrial membrane potential (MMP) between control and HD hESCs. One-Way ANOVA, ^*^p < 0.05.

Mitochondrial dysfunction comprises a robust hallmark of HD pathology, measurable by cellular dysregulation of key transcripts, deficits in mitochondrial energy capacities, and fusion/fission events (Cantuti-Castelvetri et al., 2005; Cui et al., 2006; Knott et al., 2008; Song et al., 2011b). qRT-PCR analysis of mitochondrial genes *PGC-1*α, a master regulator of mitochondrial biogenesis and energy metabolism and *DRP1*, a regulator of mitochondrial fusion and fission showed no consistent differences in transcript levels between both control and HD hESCs or neural differentiated derivatives (Figures 7A,B respectively). Intriguingly, significant (*p* < 0.05) downregulation of SI-187 *PGC-1*α mRNA levels compared to H9 samples were observed at d14 and a similar yet non-significant trend was seen with SI-186 at d14 and both HD lines at d21 compared to wildtype controls (Figure 6B). Mitochondrial functional JC-1 assays that directly measure MMP found all hESC lines demonstrated similar populations of cells exhibiting dual red and green fluorescence (HES3 95%, H9 68%, SI-186 86%, and SI-187 81%; Figure 7C), indicative of an active MMP. Remaining green fluorescent cells from each line represented a sub-population of cells with less active mitochondria.

## Discussion

Human PSCs provide a unique window to investigate the ramifications of mutant HTT within the intracellular milieu from the earliest stages of human development until the specification of relevant neuronal subtypes. This study assesses two HD hESCs carrying expanded CAG repeat lengths representative of those harbored by individuals with archetypal late clinical onset. For the first time, we report detailed quantitative evaluations of the pluripotent properties and neural differentiation capabilities of these HD hESC lines using two wildtype control hESC lines as biological comparisons. Our studies of undifferentiated hESCs show expanded CAG repeat tracts have no adverse effects on stem cell parameters that were assessed, such as viability, mitochondrial function, proliferation, and pluripotency markers. These findings are in agreement with recent pluripotent HD studies that observe no disturbance to similar parameters (Gaughwin et al., 2011; Camnasio et al., 2012; Castiglioni et al., 2012; HDIPSCC, 2012; Jeon et al., 2012).

Further, we demonstrate for the first time that neural differentiation of hESCs with HD mutations is equivalent to wildtype controls. Expression of neural markers CD56 and SOX2 and equivalent growth rates of neurospheres show unaltered differentiation in the presence of mutant HTT. Transient lag in the downregulation of the pluripotency marker CD9 was however, observed in one HD line, and corroborates findings of HD hECCs (Gaughwin et al., 2011). Crucially anterior specification assessed by key markers OTX2, FOXG1, and FORSE-1 were equivalent across all lines irrespective of CAG repeat length, and further extended differentiation produced consistent neuronal networks with similar acquisition of GABAergic identities upon *in vitro* maturation. These findings are consistent with mouse and human HD iPSC studies (Camnasio et al., 2012; Castiglioni et al., 2012; HDIPSCC, 2012).

HD patients and animal models are characterized by corticostriatal synaptic dysfunction and profligate excitotoxicity (Raymond, 2003; Estrada Sanchez et al., 2008). A major component of this pathological mechanism is glutamate receptor hypersensitivity on postsynaptic GABAergic striatal neurons. Striatal neurons receive essential glutamate signaling from cortical axons and mutant HTT is directly responsible for excessive Ca^2+^ influx downstream of glutamate stimulation, as seen in YAC128 (Tang et al., 2005) and R6/2 (Cepeda et al., 2001) transgenic models. Mutant HTT aberrantly binds endoplasmic reticulum InsP_3_R1 receptors that are critical for Ca^2+^ release. This in turn increases the responsiveness of InsP_3_R1 receptors to upstream glutamate receptor agonists and induces abnormal Ca^2+^ release (Tang et al., 2003, 2005).

In our studies we identified increased neuronal Ca^2+^ elevation in the fully penetrant HD line SI-187 upon glutamate stimulation that suggests late-onset HD hESCs are be capable of recapitulating some neuronal pathologies that characterize established HD model systems. Detectable alterations in Ca^2+^ glutamate responses are not unexpected with evidence of this phenotype in GABAergic neurons from pre-onset HD mice (Cepeda et al., 2001; Laforet et al., 2001) and even within neurons isolated as early as post-natal day 0-1 (Zeron et al., 2004). Together, these findings suggest increased Ca^2+^ responses may occur in early human stages of HD and thus represent a potentially valuable therapeutic target. The absence of such observations in the partially penetrant SI-186 line may correspond with the lesser pathological intensity of mutant proteins comprising 35–39 polyglutamine tracts and prolonged neuronal culture may be necessary to manifest this functional state.

Extensive mutant HTT dysregulation of the cellular transcriptomes is a dominant hallmark seen in post-mortem brain tissue from HD patients and across animal models, reportedly affecting ~1–2% of total cellular transcripts (Cha, 2000; Luthi-Carter et al., 2002a,b; Hodges et al., 2006). Transcript alteration has emerged as a focal point across HD pluripotent cell studies of aggressive HD subclasses, though little consensus appears to date. Exemplifying this are contrary observations of *BDNF* transcript stability and downregulation compared to controls in generated neural cultures (Gaughwin et al., 2011; Castiglioni et al., 2012; HDIPSCC, 2012). This contradiction is surprising considering clear evidence from model systems of the process by which mutant HTT instigates reduced BDNF expression via nuclear translocation of the transcriptional repressor REST (Zuccato and Cattaneo, 2007; Zuccato et al., 2007). Our study found BDNF is not altered in neural and neuronal derivatives of HD hESCs carrying typical late-onset ranges, suggesting BDNF may not be dysregulated unless significant pathology has progressed.

None of the remaining transcripts analysed in the HD hESC lines were significantly changed at either pluripotent or neural differentiated time points when compared to both control hESC lines. *SREBP1*, a cholesterol biosynthesis regulator, was significantly upregulated in pluripotent SI-187 cells compared to one control and approached significance to the second control line; a finding consistent with overt cholesterol biosynthesis disruption in HD models (Valenza et al., 2007a,b), patients (Markianos et al., 2008) and undifferentiated HD iPSC lines (Castiglioni et al., 2012). Further, there was a transient downregulation of the mitochondrial gene *PGC-1*α at d14 in SI-187 neural derivatives which continued to d21 although the trend was not statistically significant at the latter time-point. To corroborate this finding, we undertook functional studies of MMP activity, which is reduced when *PGC-1*α is down-regulated. In both undifferentiated cells and in d14 neurospheres from SI-186 and SI-187 (data not shown) we found no evidence of altered MMP. Together these findings in conjunction with those of other pluripotent systems imply transcript disturbances in this class of *in vitro* models may be a phenomenon that predominates in lines equivalent to juvenile and infantile disease subclasses, warranting array based studies to scrutinize cellular transcriptomes across a range of lines carrying different CAG repeat lengths.

In summary we have provided detailed characterization of HD hESCs and also those that harbor typical CAG repeat expansions. We demonstrate for the first time that pluripotent dynamics and forebrain neuronal differentiation are unhindered but that glutamate signaling perturbations indicative of Huntington's pathology are observable subsequent to extended *in vitro* culture and cellular acquisition of a forebrain GABAergic neuron identity. These late-onset hESC lines demonstrate the potential of such model systems to provide a platform to interrogate disease mechanisms and hallmarks. Advances describing the efficient generation of DARPP32+ medium spiny neurons provide an opportunity for probing HD etiology within a more relevant cell type (Ma et al., 2012) and could be coupled with proteasomal inhibition or oxidative stress to exacerbate disease phenotypes as demonstrated recently (HDIPSCC, 2012; Jeon et al., 2012).

Emergent studies of HD iPSCs describe lines that possess atypical alleles representative of rare juvenile/infantile (CAG_60+_) or homozygous subclasses that are poorly studied in human patients (Camnasio et al., 2012; Castiglioni et al., 2012; HDIPSCC, 2012; Jeon et al., 2012). The observation of some HD specific phenotypes in these lines demonstrate their potential value as *in vitro* human models, however, such phenotypes rarely correlate across lines and some are seen as contradictory. Existing HD iPSC lines may not faithfully represent the molecular perturbations of late-onset HD since they potentially exhibit unknown consequences by the reprogramming somatic cells exposed to mutant HTT proteins for considerable durations, and hence PGD isolated hESCs described in this study provide a more “natural” cellular alternative.

Late-onset HD alleles appear to provide a unique system to probe pre-onset changes at the molecular level and within a neurological context, as attested by observations of elevated Ca^2+^ glutamate responses. Understanding pre-onset HD alterations could elucidate the hierarchical relationship between various HD hallmarks, yet is presently restricted primarily to non-invasive imaging methodologies (Tabrizi et al., 2009; Nopoulos et al., 2011) and peripheral blood analysis (Borovecki et al., 2005; Runne et al., 2007; Bjorkqvist et al., 2008). The application of late-onset hESC to long-term *in vitro* neuronal cultures or cellular challenges that mimic *in vivo* conditions (i.e., oxidative or proteasomal stressors) may provide a system to delineate the thresholds that mark clinical onset that is otherwise difficult in extreme CAG repeat variants. Nevertheless, future work must simultaneously evaluate differing model systems to identify those which most faithfully recapitulate human HD, to steer prospective *in vitro* therapeutic activity toward models with the highest potential.

### Conflict of interest statement

The authors declare that the research was conducted in the absence of any commercial or financial relationships that could be construed as a potential conflict of interest.
